# Supplementary material for: Simple model for encoding natural images by retinal ganglion cells with nonlinear spatial integration
Source: PLoS Comput Biol. 2022 Mar 8;18(3):e1009925. doi: 10.1371/journal.pcbi.1009925 (PMC8932571; doi:10.1371/journal.pcbi.1009925)
Supplement: Attachment — Submitted filename: point_by_point_response.pdf [file pcbi.1009925.s001.pdf]

We would like to thank the two reviewers for their efforts and for the helpful, constructive feedback. In the revised manuscript, we have addressed the points raised by the reviewers as detailed in the responses below.

Reviewer #1: This paper by Liu and Gollisch studies the linearity of spatial integration in salamander RGCs. They build phenomenological models based on the average intensity in the receptive field of the cell and the spatial contrast for the cell responses and show that in many cases taking contrast into account improves performance. This provides an alternative to relatively complicated LNLN models to identify the subunits of RGC encoding. This is an interesting, yet somewhat marginal contribution. As the authors say in the Introduction, that we have long known that integration in RGCs can be nonlinear. The authors could bring out their contribution a bit more clearly.

Thanks for pointing this out. In the revised manuscript, we strive to more clearly highlight the novel contributions. First, we mention that – although nonlinear integration in RGCs has been known for long – it is not a priori clear that this translates to natural images, where contrast is typically smaller than in the reversing gratings that are typically used to asses spatial nonlinearities and where spatial correlations lead to larger regions of homogeneous contrast and emphasis on large spatial frequencies. Our work thereby contributes to the growing evidence (obtained in other species) of the importance of nonlinear spatial integration under natural stimuli. Second, and more importantly, our work highlights new methods of analyzing and quantifying the role of spatial nonlinearities under natural images and establishes a new simple modeling approach for going beyond models with single spatial filters. We now show that this approach is applicable not only to the salamander retina, but also to data from mouse retinal ganglion cells. For the latter, we have teamed up with Dimokratis Karamanlis, who had recorded appropriate data from mice and helped analyze these with the current model and who is now an additional author on the manuscript.

The maybe most important comment is that I find this paper too focused on demonstrating the models in examples cells with the population analysis getting clearly to little attention. The results section should include a section on the number of cells, what types of cells these were (mostly OFF I presume? But fast or slow?) and so on. Also indicate the example cell in all population plots.

Thanks for the suggestion. We have expanded the population analysis in the revised manuscript. In particular, we have added a clustering of the analyzed cells (which are all OFF type) into functional classes and show that spatial nonlinear effects are particularly pronounced in cells with large receptive fields, but do not differ much between cells with faster or slower kinetics. We use this also to discuss the diversity of LN model performance and of improvements found by including spatial contrast information and compare this to findings in other species. Thus, while our main focus in this work is on introducing the spatial contrast model as a tool to infer and phenomenologically capture aspects of spatial nonlinear integration, we now also emphasize the findings obtained for the salamander retina. Also, the example cells are now marked in the population plots.

Minor comments

p. 2: In fact, the bipolar cell synapse can be highly nonlinear due to the ribbon nature of the synapse and multivesicular release

Thanks. We have modified the statement and now refer to multivesicular release as a potential contribution to the nonlinear bipolar cell synapse.

p. 3: a wide range of response strengths _ was_ elicited

Thanks, corrected.

Fig. 1C-D: Why not plot Fano Factors?

We agree that Fano factors are helpful here as a commonly used measure of spike-count reliability and have added histograms of Fano factors as insets to the plots. We still want to keep the scatter plots to convey the information that images (Fig. 1C) and cells (Fig. 1D) with high Fano factor are typically those with low average responses.

p. 5: For the LSC, where the pixels also weighted by the Gaussian profile?

Yes, they were. This was perhaps a bit opaque in the writing, and we have modified the statements in both the results and methods sections to make this clearer.

p. 6: The analysis in fig. 2D-G seems fine, but overly complicated. Why not directly compare the two models with and without LSC input? That is focus on Fig. 2A and 3B.

The goal of this analysis is to directly probe and illustrate the dependence of spike counts on spatial contrast. We believe that it is instructive to see this dependence before constructing a somewhat more abstract model. An analysis as the one shown in Fig. 2F, for example, let's one read out that spatial contrast systematically increased or decreased responses for the sample cell by up to 3 spikes (beyond the response determined by mean stimulus intensity). We have revised the presentation of these analyses to make their purpose clearer.

Fig. 3C: What does "relative prediction improvement" mean?

This is the SC model performance normalized by the LN model performance, calculated as the ratio of the R^2 values from the SC model and from the LN model. We have added this information in the legend and modified the corresponding sentence in the main text for clarification.

p.7: I assume the r^2 values for the LSC model were also computed on a test set?

Yes, indeed. This is now clarified in the main text.

Fig. 3B: The improvement on the population level is obvious from the plot, but some statistics would be nice. What's the average improvement? Maybe even a statistical test.

Agreed. We now state the mean and SD of the prediction performances and have analyzed their difference with a statistical test.

More details how the LSC weight in the LSC model was fitted should be provided.

Thanks for picking this up. Some information was missing here. We now point out that the weight was obtained by a least-squares fit that was repeatedly alternated with the least-squares fit of the nonlinearity. We have also corrected the corresponding section in Methods; the nonlinearity was fit directly on the 150 responses to the natural images, not on histogram values, as previously stated.

Could the authors speculate why some neurons behave more linearly (little improvement by adding LSC) and some more non-linearly? Could that be related to different cell types (see below)? Are the cells with little performance gain by LSC tuned to high frequencies?

To address this question, we have aimed at dividing the analyzed cells into functional classes (see also response to comment below) and indeed found that it is cells with larger receptive field whose response prediction benefits more from including spatial contrast information. These results are presented in the new Fig. 4, and they indicate that the diversity of prediction improvements indeed depends on cell type, similarly to findings in other species. In the Discussion, we now also pick up the diversity of response predictions and the relation to cell types in the present and in other works.

Suggestions for additional analysis that would add to the paper:

Does the cell type of the cell (ON/OFF, fast/slow, …) matter for the improvement in predictive performance and sensitivity for local contrast? Especially the analysis in Fig. 4F would be interesting to see by cell type.

Thanks for the suggestion. As already mentioned above, we now include a classification of the analyzed ganglion cells into four groups and present the analysis in the new Fig. 4. This indeed indicated that cell type likely matters for the prediction improvement through spatial contrast, as the classes of large cells benefited most from this information. Note that we restricted our analysis to OFF ganglion cells, as these constitute the vast majority of salamander ganglion cells. ON cells were only rarely encountered in our recordings, and ON-OFF cells would require a more refined treatment of the two input pathways and the nonlinear output function. This is now also more clearly stated in manuscript.

Would the authors assume that this also works for e.g. mouse RGCs? Do they have data that could be used to show it?

Thanks for the suggestion. We now include analyses of mouse ganglion cells, comparing the classical LN and the spatial-contrast model. As mentioned above, we teamed up with Dimokratis Karamanlis for this, who had recorded appropriate data from mice and helped analyze these with the current model and who is now an additional author on the manuscript. The results are shown in the new Fig. 5. Findings are overall similar to the analysis of salamander ganglion cells. A correlation between differences in spike count and differences in spatial contrast for image pairs with similar mean luminance information in the receptive field indicates the importance of spatial contrast information. And the spatial contrast model often yields better spike count predictions than the LN model. These results underscore that the spatial contrast model is generally applicable to different model systems.

It would be interesting to see how the two models discussed here compare against a standard LN

model (with fitted linear filter) and a LNLN model fitted based on the data using efficient inference techniques.

This is an interesting suggestion. Please note, though, that one of the two models is already a standard LN model, with the linear filter fitted to the STA obtained from white-noise experiments. Fitting the filter to the responses under natural images is, unfortunately, not feasible because of the few applied images and the spatial correlations of the stimulus. Comparing the models to predictions from LNLN cascades is certainly an interesting endeavor. However, fitting LNLN models to data is still a tremendous challenge with no general solution. Although some methods have been proposed to obtain the subunits of the first linear-filter stage (including our own spike-triggered non-negative matrix factorization), this requires dedicated long experiments (with white-noise stimulation), which are not available for the current data. Also, turning the subunits into full-fledged LNLN models (e.g. with optimized subunit nonlinearities and optimized regularization) is not fully solved, and exploring this would be beyond the scope of the current manuscript. We hope, on the other hand, that the current spatial-contrast model will be useful for developments of fitted LNLN models, and we plan to include such a comparison in future investigations. We have included some of these points in the Discussion.

Reviewer #2: This work starts from the well-known, but hard-to-model fact that retinal ganglion cells display nonlinear spatial summation of their input subunits. In current work, this can be modeled by either 1) measuring the subunit complement for each RGC directly through extensive data collection with small stimulus patches or 2) exploring a best-fit quadratic model (instead of a linear receptive field model) that also requires large amounts of data. Here, the authors propose using a particular quadratic function, the standard deviation of input intensities within the receptive field, or local spatial contrast (LSC). The manuscript shows that incorporating LSC into a standard linear-nonlinear Poisson model captures significantly more of the spiking response in salamander RGC's. By smoothing the input image, spatially and systematically, the authors show that the optimal spatial scale corresponds to about a third of the RGC receptive field size, which roughly aligns with bipolar subunit receptive field sizes in the salamander.

Overall, the work is clear, clearly presented, and clearly correct. The paper is well-written and thoroughly referenced and provides a useful tool for experimentalists to "level up" in their modeling of RGC's beyond typical, but known to be poor performing, LNP models.

The optimal spatial scale calculation in Figure 4 seems to have weak explanatory power across the cell population and one wonders whether the absolute scale quoted is statistically significant. Is any of this improved by separating by RGC sub-types in the analysis?

Thanks for the suggestion. We now include a separation of the salamander ganglion cells into functional classes by separating the cells (via a cluster analysis) according to receptive field size and temporal filtering kinetics. Using this classification, we now show that it is the classes with larger receptive fields that are affected most by spatial nonlinearities and by information about spatial contrast but that their optimal spatial scales do not differ from those of smaller cells. This suggests, for example, that larger cells rather have more than larger subunits. The results are presented in the new Figure 4 and in new panels of what is now Figure 6. We believe that this considerably enhances the results and highlights the potential of the presented methods and model.

There is one major concern that would be worth addressing:
The LSC choice seems like a good guess at what might be a baseline relevant features for accounting for spatial nonlinearities in RGCs. However, as the authors note in the discussion, one could have used a generalized quadratic function to fit the RGC receptive field. It would be extremely useful to fit this kind of model for at least a subset of the data and show that the LSC was, indeed, the right best guess, by comparing performance between models.

We agree that comparison with an actual fitted generalized quadratic model would be an interesting comparison. However, these models are very data-hungry and require dedicated long recordings, especially in the case when the input is high-dimensional, as is the case when spatial or spatiotemporal stimuli are considered. We have tried with a few sample cells where responses to spatiotemporal white noise were available, but these efforts have suffered from problems with convergence and overfitting issues, owing to the discrepancy between the number of parameters in the generalized quadratic model and the amount of available data. We therefore leave this endeavor for a future dedicated study. These issues are now also mentioned in the Discussion.

Minor comment: It would be nice to include N's (156) in Figure 1 for the number of cells used in the analysis and to include a note in the caption saying how many different retinas (9?) these N cells come from, instead of just having that info in the Methods.

Thanks, done.
